# PRIORI-T: A tool for rare disease gene prioritization using MEDLINE

**DOI:** 10.1371/journal.pone.0231728

**Published:** 2020-04-21

**Authors:** Aditya Rao, Thomas Joseph, Vangala G. Saipradeep, Sujatha Kotte, Naveen Sivadasan, Rajgopal Srinivasan

**Affiliations:** TCS Research and Innovation, Tata Consultancy Services Ltd., Hyderabad, INDIA; HudsonAlpha Institute for Biotechnology, UNITED STATES

## Abstract

**Introduction:**

Phenotype-driven rare disease gene prioritization relies on high quality curated resources containing disease, gene and phenotype annotations. However, the effectiveness of gene prioritization tools is constrained by the incomplete coverage of rare disease, phenotype and gene annotations in such curated resources.

**Methods:**

We extracted rare disease correlation pairs involving diseases, phenotypes and genes from MEDLINE abstracts and used the information propagation algorithm GCAS to build an association network. We built a tool called PRIORI-T for rare disease gene prioritization that uses this network for phenotype-driven rare disease gene prioritization. The quality of disease-gene associations in PRIORI-T was compared with resources such as DisGeNET and Open Targets in the context of rare diseases. The gene prioritization performance of PRIORI-T was evaluated using phenotype descriptions of 230 real-world rare disease clinical cases collated from recent publications, as well as compared to other gene prioritization tools such as HANRD and Orphamizer.

**Results:**

PRIORI-T contains qualitatively better associations than DisGeNET and Open Targets. Furthermore, the causal genes were captured within Top-50 for more than 40% of the real-world clinical cases and within Top-300 for more than 72% of the cases when PRIORI-T was used for gene prioritization. It outperformed other gene prioritization tools such as HANRD and Orphamizer that primarily rely on curated resources.

**Conclusions:**

PRIORI-T exhibited improved gene prioritization performance without requiring high quality curated data. Thus, it holds great promise in phenotype-driven gene prioritization for rare disease studies.

## 1 Introduction

One of the major challenges in genomic medicine, especially for rare diseases, is the identification of causal genomic variants by establishing their relationship to the observed clinical phenotypes of patients [1]. Known associations between various biomedical entities such as genes, diseases and phenotypes from curated resources such as Orphanet [2] are crucial for discovering novel phenotype-genotype and for disease gene prioritization. However, structured resources suffer from low coverage and from not being up to date, often because the curation effort is both time and labor-intensive [3, 4]. A significant fraction of known associations is mentioned only in unstructured biomedical literature [5], implying a need for gene prioritization tools that make use of associations extracted using text-mining.

Computational deep phenotyping is now considered an important aid in the analysis of genomic data for personalized genomic medicine. Tools such as Phenomizer [6], Orphamizer (a version of Phenomizer that uses Orphanet) [6] and HANRD [7] use a list of phenotype terms as input to find potential candidate diseases and their corresponding causal genes. These tools use associations from curated resources such as Orphanet, HPO [8] and OMIM [9], amongst others. Tools such as Beegle [10] extract disease-gene associations from MEDLINE for disease-gene prioritization. Such tools focus mainly on extracting disease-gene associations from literature. However, Beegle is broad-based and lacks focus on rare disease associations. Previous studies have suggested that use of a rare disease-centric corpus as well as specialized search are better than more generic databases and search tools in rare disease diagnosis [11, 12]. The potential of using text-mining of disease-phenotype associations from clinical case reports as a means of improving the performance of phenotype-driven differential-diagnosis systems for rare diseases has been reported [13].

We try to address these challenges by building a rare disease gene prioritization tool called PRIORI-T. PRIORI-T uses a comprehensive set of correlation pairs such as disease-gene, phenotype-phenotype and phenotype-disease extracted from a corpus of rare disease MEDLINE abstracts for gene prioritization. We have previously described TPX, a web-based text-mining tool that supports real-time entity assisted search and navigation of the MEDLINE repository whilst continuing to use PubMed as the underlying search engine [14]. The dictionary-based named entity recognition (NER) module of TPX was repurposed and used in PRIORI-T for identifying entities such as rare diseases, phenotypes and genes in MEDLINE abstracts. Pairwise correlations were computed between these co-occurring entities using Pearson correlation coefficient and an initial correlation network (ICN) was constructed using these correlations.

However, such a network is bereft of indirect associations that could link nodes not directly connected to each other. We used the information propagation algorithm GCAS (*Graph Convolution-based Association Scoring*) [7] to propagate information across the correlation pairs in the ICN to infer indirect associations. The ICN was augmented with these inferred associations and the resulting network is referred to as *association network* (ASN). The ASN was used by PRIORI-T’s Gene Prioritization module for rare disease gene prioritization. The input to PRIORI-T is a list of phenotypes obtained from a clinical case and the output a ranked list of genes that could possibly contain the causal gene(s). PRIORI-T was evaluated using the curated disease data from Orphanet using the associated causal gene information, as well as text-mining based resources such as BeFree [5] and Open Targets [15]. It was also evaluated on 230 real-world clinical cases collated from recent publications [16, 17, 18, 19].

## 2 Materials and methods

**Fig 1:**
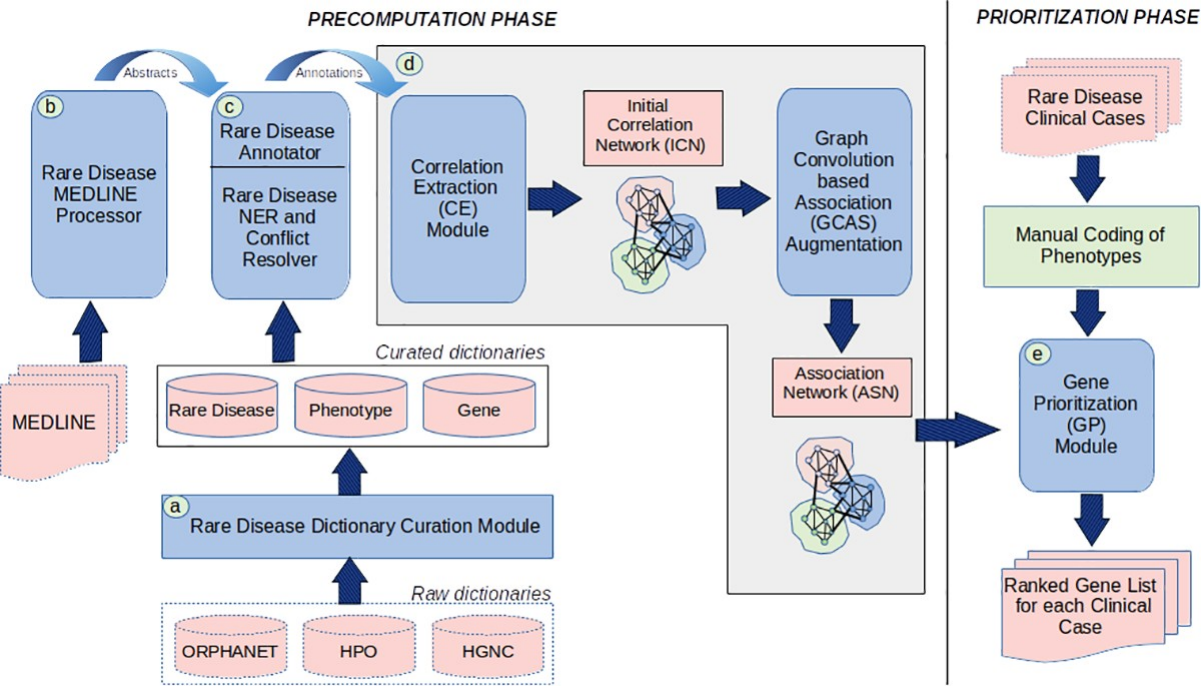
PRIORI-modules: (a) Rare Disease Dictionary Curation module, (b) Rare Disease Annotator (c) Rare Disease MEDLINE Processor (d) Network Creation module (e) Gene Prioritization module.

In this section, we first describe the modules of PRIORI-T (Fig 1): (a) Rare Disease Dictionary Curation module, which creates rare disease specific dictionaries from resources such as HPO, Orphanet and HGNC; (b) Rare Disease Annotator to identify entities relevant to rare diseases using the above dictionaries; (c) Rare Disease MEDLINE Processor, where MEDLINE abstracts pertinent to rare diseases were processed and annotated for diseases, phenotypes and genes; (d) Network Creation module, which uses the Correlation Extraction (CE) module to construct an initial correlation network (ICN), and further augments this ICN using the inference algorithm GCAS to construct the final association network (ASN); and (e) Gene Prioritization module, which performs phenotype driven gene prioritization using the ASN. Modules (a)-(d) described above are part of the precomputation phase, while (e) is the gene prioritization phase.

We then evaluate the quality of associations in PRIORI-T against data from curated and text-mining resources. Finally, we performed gene prioritization using PRIORI-T on a dataset of 230 solved rare disease clinical cases compiled from four recent publications.

### 2.1 Modules of PRIORI-T

#### a) Rare disease dictionary curation

We created human rare disease domain-specific dictionaries using HPO [8], Orphanet [2] and HGNC [20] as the main data resources. We augmented the dictionaries using text patterns as well as MeSH mappings provided by Orphanet and HPO. The dictionaries for phenotypes, rare diseases and genes were created using HPO (accessed May 7, 2019), Orphanet (accessed June 27, 2019) and HGNC (accessed May 7, 2019) respectively. The terms from Orphanet and HPO were also augmented by corresponding MeSH terms obtained using their MeSH mappings. Manual inspection yielded some missing synonyms that were then added to the dictionary.

We observed issues of overlaps, ambiguity and coverage, both within a particular dictionary type and across dictionary types [Table 1]. To address these issues, we used a semi-automated dictionary curation process to build individual dictionaries [21].

**Table 1:** Examples of overlaps within and across dictionaries.

Overlap Type	Entity	ID1	ID2
HGNC	cox2	HGNC:7421	HGNC:9605
	nat3	HGNC:14679	HGNC:15908
Orphanet-HPO	submucosal cleft palate	ORPHA:155878	HP:0000176
	fulminant hepatic failure	ORPHA:90062	HP:0004448
HGNC-HPO	Paroxysmal nocturnal hemoglobinuria	HGNC:8957	HP:0004818
	Warts	HGNC:6514	HP:0200043
HGNC-Orphanet	cayman ataxia	HGNC:779	ORPHA:94122
	spg71	HGNC:17277	ORPHA:401840

Overlaps within a dictionary were disambiguated in a semi-automated manner, while entities that overlapped across dictionaries were manually disambiguated. The remaining entities were left as-is in the dictionaries and resolved using the Rare Disease Conflict Resolver module described later below. Furthermore, noisy acronyms and high-level entities were removed after analyzing the frequencies of their occurrences across MEDLINE. The term counts of disease, phenotype and gene dictionaries before and after the curation are shown in [Table 2].

**Table 2:** Dictionary term counts before and after curation.

Dictionary	Main Resource	Additional Resource(s)	Before Curation	After Curation
Disease	22,546	20,294	42,840	45,856
Phenotype	30,812	17,915	48,727	55,183
Gene	80,832	5	80,832	82,387

#### b) Rare disease MEDLINE processor

The rare disease MEDLINE processor first fetches all MEDLINE abstracts (29 million abstracts). In order to consider only those MEDLINE abstracts that were relevant to rare diseases, it then filters out abstracts that did not contain at least one Orphanet rare disease term. It obtained 2.4 million abstracts containing at least one rare disease term which were considered for analysis. This acts as a means of using only those abstracts that have a rare disease context.

#### c) Rare disease annotator

The Rare Disease Annotator comprises of 1) NER module that annotates rare disease abstracts, from the MEDLINE processor using the rare disease dictionaries, and 2) Conflict Resolver (CR) module that performs entity disambiguation by resolving conflicts between multiple annotations. These modules were repurposed from the TPX text-mining framework.

The NER module from TPX was repurposed to identify phenotype, rare disease and gene term mentions in the text using the curated rare disease dictionaries described above. To separately handle each rare disease entity type, individual NER modules were developed. For instance, rare disease terms in the text are tokenized and matched differently compared to gene terms. Additionally, the NER module uses separate entity specific stop word lists for removing stop words corresponding to each dictionary type. The CR module then resolves the conflicts due to overlapping entity annotations. The CR uses a simple entity disambiguation rule—”Phenotype *wins over* Disease *wins over* Gene” to resolve conflicts across these entity types. We found that 95.5% of the conflicts across entity types resolved by CR were phenotype-disease while 2.2% and 2.3% were phenotype-gene and disease-gene conflicts respectively. The Rare Disease Annotator identified 6282 diseases, 8043 phenotypes and 14,430 genes in these 2.4 million abstracts.

#### d) Construction of initial correlation network (ICN) and association network (ASN)

The Correlation Extraction module uses the Pearson correlation coefficient to compute pairwise correlations between entities identified by the Annotator. To compute the pair-wise correlation for the pair (a, b), we used the standard Pearson correlation expression

$$\rho_{a,b}=\frac{P(a,b)-P(a)P(b)}{\sqrt{P(a)P(b)(1-P(a))(1-P(b))}}$$

where P(a, b) denotes the joint probability of co-occurrence of entities a and b. Terms P(a) and P(b) denote the marginal probabilities of occurrences of entities a and b respectively. These probability values are estimated from the corpus.

We computed pair-wise correlations both at the sentence level and at the abstract level. Based on our evaluations, we decided to include only the correlations computed at the abstract level to build the initial correlation network. The network built using these pairwise correlations constitutes the Initial Correlation Network (ICN). The entities are the nodes of this network and the pair-wise correlations are represented using undirected weighted edges where the edge weight corresponds to the correlation strength.

Relying only on these direct associations in the ICN can be a limiting factor for tasks such as gene prioritization. Hence, we used the information propagation algorithm GCAS (*Graph Convolution-based Association Scoring*) [7] that uses graph convolution to propagate information between unconnected entities in a network in order to mine indirect associations between these entities.

GCAS uses graph convolution to propagate information between entity pairs in a network and uses this propagated information to assign association scores between entity pairs which are not connected by direct links. Information propagation on a network consisting of n nodes is viewed as propagation of an n-dimensional signal x on the network corresponding to the graph G, which in turn corresponds to convolving the signal x with a filter g. Using methods from graph signal processing and spectral graph theory [22], this can be expressed as a point-wise multiplication of the graph Fourier transforms of the signal and the filter computed with respect to the given graph G. The goal then to design a filter g that achieves the desired signal propagation on G. GCAS uses first-order approximation of the convolution with a reduced parameter space. Suitable choices for the parameters are computed based on its performance on random sub-networks. Under this simplified model, the information propagated to the t-th order neighbor of a node is obtained by t consecutive application of the one-step convolution.

The ICN augmented with the inferred associations computed using GCAS is referred to as the Association Network (ASN).

#### e) Gene prioritization module

The ASN is used by the gene prioritization module of PRIORI-T for rare disease gene prioritization. The input to this module is a list of phenotypes from a clinical case. These are used to search the ASN and come up with an output list of ranked genes.

### 2.2 Quality evaluation of PRIORI-T associations

A pragmatic way to assess the quality of associations in PRIORI-T is to compare it against curated [5] as well as text-mining resources. PRIORI-T was compared with the curated Orphanet disease-gene associations as follows: For each disease in Orphanet, the ROC (Receiver Operating Characteristic) curve and its AUC (Area Under the Curve) score [23] were computed by looking at the ranks of the known genes associated with the disease amongst all its gene neighbors in PRIORI-T. Gene neighbors are the direct neighbors of the disease in the PRIORI-T network (ASN). In AUC_*N*_, the ROC curve is computed with respect to the relative ordering of all the true gene neighbors and the first N false neighbors within the given ordering. AUC_*N*_ essentially corresponds to the probability that, in a given ordering, a randomly picked true neighbor appear before a randomly picked false neighbor from among the first N false neighbors in the ordering. As a consequence, AUC_*N*_ < = AUC_Overall_ for all N. The average AUC_Overall_ and AUC_*N*_ scores for PRIORI-T were plotted. We also evaluated the ICN and ASN associations by comparing with Orphanet associations. Additionally, we compared the correlations derived from a sentence with those derived from the entire abstract.

We also evaluated DisGeNET and Open Targets data, where, for each disease term, the gene(s) from these datasets are compared against Orphanet. Towards this, the ROC score was computed by looking at the ranks of the genes associated with each Orphanet disease amongst all its gene neighbors in DisGeNET_*BeFree*_ and Open Targets_*Literature*_ respectively. DisGeNET_*BeFree*_ refers to the rare disease related DisGeNET associations extracted using the BeFree tool, and Open Targets_*Literature*_ refers to the rare disease related text-mining-derived Open Targets associations. The AUC_*N*_ value of the ROC curve of DisGeNET_*BeFree*_ and Open Targets_*Literature*_ were plotted against Orphanet disease-gene associations. Additionally, we included all rare-disease related disease-gene associations from DisGeNET and Open Targets instead of only the text-mining associations and plotted the AUC_*N*_ value of the ROC curve.

### 2.3 Gene prioritization using PRIORI-T

PRIORI-T was used for rare disease gene prioritization on a dataset of 230 solved rare disease real-world clinical cases compiled from four recent publications [16, 17, 18, 19]. This dataset (S1 File) had a list of clinical phenotype terms for each case, the diagnosed disease(s) and the identified causal gene(s), amongst other fields. We used the Rare Disease Annotator to assign HPO codes to the clinical phenotype terms. However, for those clinical phenotype terms which couldn’t be coded by our annotator, we manually assigned appropriate HPO codes. We corroborated all the HPO codes before using them in the gene prioritization module. For each case, the input query to PRIORI-T gene prioritization module was its HPO IDs representing the clinical phenotype and the output is a ranked list of genes associated with the input. To avoid any bias, we excluded the four publications and their associated 329 citations from the corpus of MEDLINE abstracts used in PRIORI-T. The percentage of input cases where the causal gene appeared within a Top-*k* of this ranked list of genes was computed.

We additionally compared the Top-*k* ranks for these 230 clinical cases against other gene prioritization tools such as HANRD and Orphamizer. The percentage of input cases where the causal gene appeared within a Top-*k* of this ranked list of genes was computed for each of these tools. Furthermore, we included two additional variants of PRIORI-T to study the impact of curated associations and inferred associations in the gene prioritization task. The first is a hybrid network of curated associations from HANRD along with the ICN. In this network, we considered the association score as-is if the association exists in either one but not in the other. We also considered the score of the curated association from HANRD if there is an overlap of edges. GCAS was run on this hybrid network to infer associations and the PRIORI-T(HANRD_INITIAL_+ICN) instance created. Gene prioritization was performed using PRIORI-T(HANRD_INITIAL_+ICN) and Top-*k* ranks were computed. The second PRIORI-T variant network consisted of only the ICN in PRIORI-T to create a PRIORI-T_ICN_ instance and the Top-*k* ranks were computed. This was done to assess the significance of the inferred associations.

## 3 Results

A total of 3.45 million (3,452,672) correlated pairs were computed from the 2.36 million (2,35,9264) MEDLINE abstracts. Negatively correlated pairs were excluded from this, resulting in a total of 2.9 million (2,937,220) correlated pairs. The ICN is built using these correlation pairs, where each edge corresponds to a correlated pair and the edge weight is the Pearson score. S1 File shows the counts of the different correlation pair types in the ICN. S1 File shows the counts of the different correlation pair types in the ASN. The ASN is the default network used by PRIORI-T in this study.

### 3.1 Quality evaluation of PRIORI-T associations

**Fig 2:**
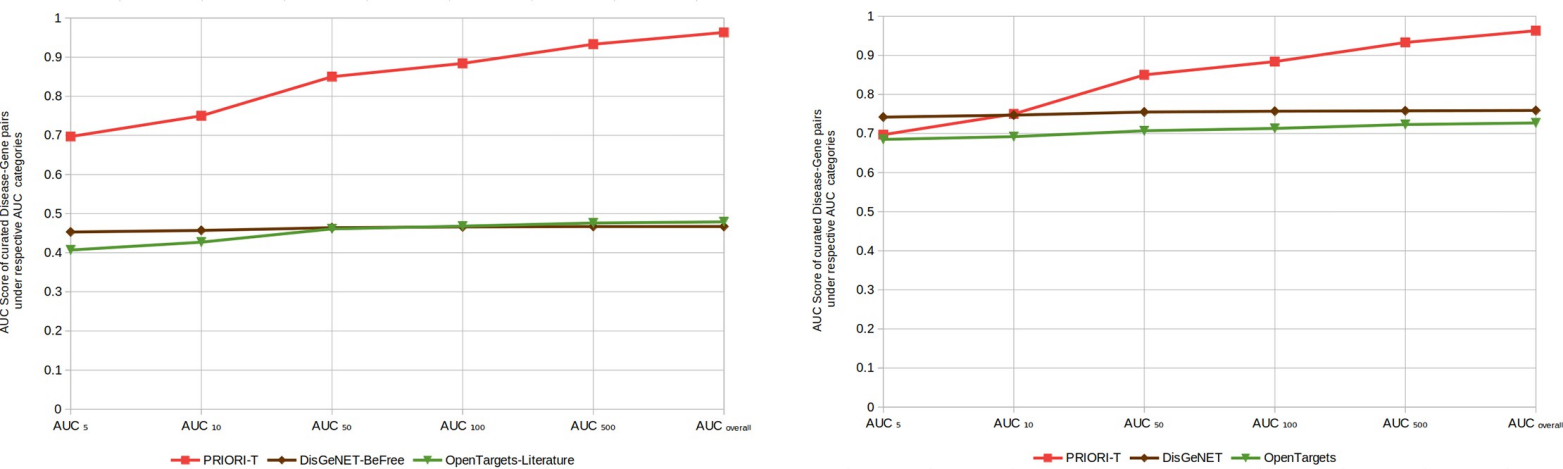
(a) Comparison of the quality of associations of PRIORI-T with DisGeNET_*BeFree*_ and Open Targets_*Literature*_ using Orphanet associations. (b) Comparison of the quality of associations of PRIORI-T with rare-disease related DisGeNET and Open Targets using Orphanet associations.

Fig 2A shows the AUC_*N*_ comparison of PRIORI-T against Orphanet disease-gene associations. For each disease in Orphanet, the ROC score was computed by looking at the ranks of the linked genes associated with the disease amongst all its gene neighbors in PRIORI-T. As seen in the figure, PRIORI-T achieved an AUC score of 87% in Top-50, and an overall AUC of more than 94% respectively. Thus, PRIORI-T exhibited better AUC when compared against Orphanet. Fig 2A also shows the AUC_*N*_ value for DisGeNET_*BeFree*_ and Open Targets_*Literature in*_ comparison to Orphanet disease-gene associations. As seen in the figure, PRIORI-T had better AUC score when compared DisGeNET_*BeFree*_ and Open Targets_*Literature*_. Similarly, Fig 2B shows the AUC_*N*_ value of the ROC curve of rare disease specific DisGeNET and Open Targets when compared to Orphanet disease-gene associations. As seen, PRIORI-T had improved AUC scores when compared to the text-mining specific DisGeNET and Open Targets as well as the complete DisGeNET and Open Targets associations.

### 3.2 Rare disease gene prioritization using PRIORI-T

**Fig 3:**
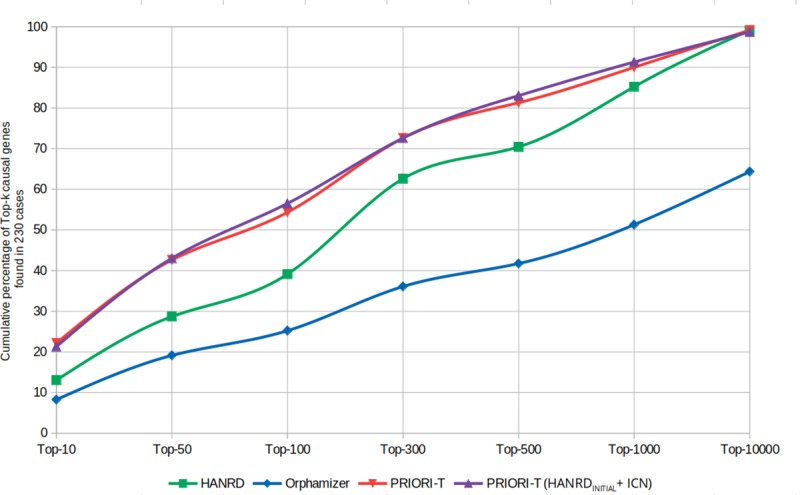
The cumulative percentage of causal genes found in Top-k when gene prioritization was performed on 230 real-world rare disease clinical cases using HANRD, PRIORI-T, PRIORI-T(HANRD_INITIAL_+ICN) and Orphamizer.

PRIORI-T was used for rare disease gene prioritization on a dataset of 230 real-world rare disease clinical cases compiled from four recent publications. Fig 3 shows the Top-k distribution of the causal genes on the 230 rare disease clinical cases from these publications. As seen here, PRIORI-T captured causal gene(s) for more than 40% of the cases in Top-50 and more than 72% of the cases in Top-300. In comparison, HANRD captured 30% and 62% respectively. Thus, PRIORI-T ranks the known causal gene for most of the cases higher than HANRD, despite HANRD having the benefit of curated associations from Orphanet. Both PRIORI-T and HANRD performed better than Orphamizer. Gene prioritization was also performed on the combined network PRIORI-T(HANRD_INITIAL_+ICN) constructed using the curated associations of HANRD and the ICN. As seen in the figure, there is no significant improvement in the performance of the combined approach over PRIORI-T because ICN itself accounted for most of the curated associations. This shows that correlation networks can provide competing performance when compared to high quality curated resources. Furthermore, using PRIORI-T on the default ASN identified causal gene(s) for 8 more cases than PRIORI-T_ICN_, indicating an improved recall when inferred associations are included. Thus, PRIORI-T on the default ASN achieved a performance which was comparable to that of PRIORI-T(HANRD_INITIAL_+ICN) without using any curated data resources. Thus, PRIORI-T, containing only textual correlations and inferred associations suffices for the task of gene prioritization.

**Fig 4:**
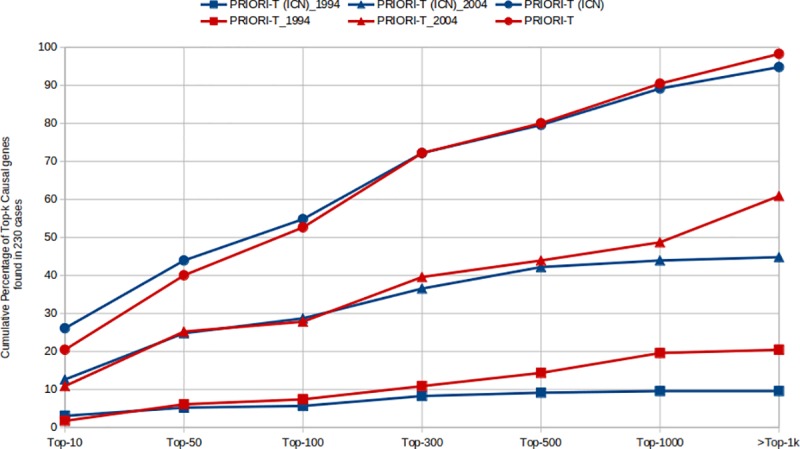
The Top-k distribution of the causal genes plotted for each the three time-series datasets–up to 1994, 2004 and 2018 for both ICN and ASN.

To explore the effect of inferred associations on recall, MEDLINE abstracts published until 1994, 2004 and 2018 (as of June 1^st^, 2018) respectively were used to create three datasets of abstracts. We performed the prioritization task by using PRIORI-T on the ICN and ASN and plotted the results (Fig 4). We observed that the inferred associations of ASN contributed to a significant improvement in Top-k recall after Top-100 for the 1994 and 2004 datasets. However, the inferred associations of ASN had lesser effect on recall for the 2018 dataset. The 1994 and 2004 datasets had relatively smaller number of abstracts related to these 230 cases than the 2018 dataset and therefore the ICN had lower number of relevant correlation pairs. Thus, inferred associations are able to improve the overall prioritization performance when the correlation pairs from the published literature are limited.

## 4 Discussion

We built the PRIORI-T tool for phenotype-driven rare disease gene prioritization which uses an input list of phenotypes that describe a clinical case. The modules of PRIORI-T included a Rare Disease Dictionary Curation module, a Rare Disease Annotator for rare disease-related entity annotation, the Rare Disease MEDLINE Processor, a Network Creation module to build the ASN network combining the correlations extracted by the Correlation Extraction module and GCAS inferred associations, and finally the Gene Prioritization module where genes are prioritized using the ASN.

The Rare Disease Dictionary Curation module required novel rare disease-specific dictionaries to be built. Conflicts and overlaps amongst these dictionaries had to be resolved before they could be used for NER in the Rare Disease Annotator module. This module identifies rare disease-specific term mentions in the entire MEDLINE corpus. It has been shown that specialized query terms are required to identify such rare disease mentions in MEDLINE [24]. Augmenting the dictionaries with such specialized terms might improve the NER module coverage. Another approach could be to use machine learning or deep learning based NER techniques to better identify such disease term mentions [25].

MEDLINE abstracts relevant to rare diseases were identified by the Rare Disease MEDLINE Processor module using terms from the disease dictionary. Given that the mention of a rare disease term doesn’t necessarily mean the abstract is about that disease, extraneous abstracts are also selected. Instead of this approach, we also considered using MeSH disease annotations as a limiting parameter in the Entrez Programming Utilities (E-utilities). However, incomplete MeSH disease annotations of PubMed abstracts prevented us from using it in this study. In the future, we intend to explore this option as a means of selecting rare disease MEDLINE abstracts. Some phenotype terms will co-occur with genes in many MEDLINE abstracts. Hence, these associations might not be of much informational value. Some phenotype-gene correlations could also have been the filtered out when filtering abstracts based on presence of a rare disease.

Using Pearson correlation coefficient, we computed pairwise correlations between entities identified by the Annotator at both sentence and abstract levels. Abstract-level associations had a slightly better AUC score than those derived from a sentence level (S1 File). This could possibly due to a higher recall resulting from using the abstract level associations and a comparable precision because the final ranking of the associations is controlled by their Pearson correlation strengths. These correlation pairs form what is known as the initial correlation network (ICN). We then used the information propagation algorithm GCAS (Graph Convolution-based Association Scoring) to propagate information between unconnected entities in a network in order to establish indirect associations between these entities. The ICN augmented with inferred associations is referred to as the Association Network (ASN). ASN showed a better AUC score compared to the ICN correlations (S1 File). Hence, in this study, the ASN is the default network used by PRIORI-T.

We compared the associations in PRIORI-T with curated associations from Orphanet. PRIORI-T had an AUC score of 87% in the top 50. One of the reasons some of the Orphanet associations are missing in PRIORI-T could be that Orphanet curators rely on comprehensive associations found in full-text articles, rather than only on abstracts, which have limited coverage. Extracting correlation pairs from full-text repositories such as PubMed Central might help in capturing some of these ‘missing’ associations. Another reason could be due to the untagged disease term mentions as discussed above. The PRIORI-T associations also contained several novel association pairs that were not present in Orphanet (some examples are shown in S1 File). While these pairs look promising from a manual inspection, these need a careful evaluation to filter out any false positives. These could either be due to abstracts not being relevant to rare diseases getting included or the CE module extracting associations that are negatively or speculatively associated. Thus, it is clear that only after comprehensive analysis can associations in PRIORI-T be considered for addition to resources such as Orphanet. PRIORI-T also showed better AUC values when compared to the text-mining specific DisGeNET and Open Targets as well as the complete DisGeNET and Open Targets associations.

Gene prioritization using PRIORI-T was evaluated on 230 clinical cases where it was seen that the causal genes for 40% of these cases were captured in the Top-50 and 72% in the Top-300. This is despite the low number of phenotypes as well as their generic nature and their limited discriminative power in many of the 230 clinical cases, Moreover, PRIORI-T was seen to outperform tools such as HANRD and Orphamizer. PRIORI-T relies on correlations extracted from MEDLINE abstracts while our previously described tool HANRD is made up of curated and ontological associations. However, the GCAS algorithm used to generate the inferred associations is the same in both tools. Despite lacking curated or ontological associations, the PRIORI-T tool outperformed the HANRD network in the rare disease gene prioritization task. In fact, addition of curated associations to PRIORI-T showed no advantage over text-mined associations. This could be possibly due to the availability of more correlations derived from text-mining when compared those from curated resources. We also found an improved recall when inferred associations are added to the MEDLINE-derived correlations in PRIORI-T.

The lack of availability of high quality and heterogeneous curated data with sufficient coverage can affect the success of gene prioritization tools that rely primarily on curated resources such as Orphanet and HPO. Moreover, constructing such curated resources is manually intensive. Hence, we took a different approach where our prioritization tool PRIORI-T relied on MEDLINE-derived correlations and their inferred associations. PRIORI-T performed better on real world cases compared with tools using curated data and was able to identify the causal genes with high ranks. Combining a variant prioritization tool with PRIORI-T might help in a more accurate identification of causal genes in rare disease clinical cases.

## Supporting information

S1 File The supporting file contains list of clinical phenotype terms for each of the 230 cases along with the diagnosed disease(s) and the corresponding causal gene(s). It also contains all the supplementary tables and figures.
(PDF)
